# Differential Effects of Dietary Components on Glucose Intolerance and Non-Alcoholic Steatohepatitis

**DOI:** 10.3390/nu13082523

**Published:** 2021-07-23

**Authors:** Josephine Skat-Rørdam, David Højland Ipsen, Patrick Duncan Hardam, Markus Latta, Jens Lykkesfeldt, Pernille Tveden-Nyborg

**Affiliations:** 1 Section of Experimental Animal Models, Department of Veterinary and Animal Sciences, Faculty of Health and Medical Sciences, University of Copenhagen, 1870 Frederiksberg, Denmark; jsr@sund.ku.dk (J.S.-R.); dhi@sund.ku.dk (D.H.I.); patrickdh@sund.ku.dk (P.D.H.); jopl@sund.ku.dk (J.L.); 2 Liver Disease Research, Global Drug Discovery, Novo Nordisk A/S, 2880 Copenhagen, Denmark; mrlq@novonordisk.com

**Keywords:** NASH, glucose intolerance, diet, starch, soft drink

## Abstract

Pharmacological treatment modalities for non-alcoholic fatty liver disease (NAFLD) and steatohepatitis (NASH) are scarce, and discoveries are challenged by lack of predictive animal models adequately reflecting severe human disease stages and co-morbidities such as obesity and type 2 diabetes. To mimic human NAFLD/NASH etiology, many preclinical models rely on specific dietary components, though metabolism may differ considerably between species, potentially affecting outcomes and limiting comparability between studies. Consequently, understanding the physiological effects of dietary components is critical for high translational validity. This study investigated the effects of high fat, cholesterol, and carbohydrate sources on NASH development and metabolic outcomes in guinea pigs. Diet groups (*n* = 8/group) included: low-fat low-starch (LF-LSt), low-fat high-starch (LF-HSt), high-fat (HF) or HF with 4.2%, or 8.4% sugar water supplementation. The results showed that caloric compensation in HF animals supplied with sugar water led to reduced feed intake and a milder NASH phenotype compared to HF. The HF group displayed advanced NASH, weight gain and glucose intolerance compared to LF-LSt animals, but not LF-HSt, indicating an undesirable effect of starch in the control diet. Our findings support the HF guinea pig as a model of advanced NASH and highlights the importance in considering carbohydrate sources in preclinical studies of NAFLD.

## 1. Introduction

Non-alcoholic fatty liver disease (NAFLD) and the more advanced form steatohepatitis (NASH) are multifactorial and progressive diseases, and in humans are often accompanied by comorbidities such as obesity, insulin resistance and dyslipidemia [1]. Reflecting the global ‘obesity-pandemic’, the incidence of NAFLD is rapidly increasing to become the most common liver disease worldwide, estimated to affect up to 25% of the adult population [2]. The deposition of surplus fat in hepatocytes (hepatic steatosis) represents the initial hallmark of the disease and is accelerated by an excessive energy consumption. This is in line with the association with obesity and metabolic dysfunction reported for many human patients and commonly linked to a diet rich in fat, cholesterol and carbohydrates [3,4]. However, the complexity of human NAFLD/NASH has proven difficult to mirror experimentally and animal models accurately recapitulating the disease etiology and phenotype are scarce, reducing the predictive validity of findings and limiting research advances [5,6]. 

Despite recommendations of a maximum intake of 10%, the average American consumes around 17% of their calories from added simple carbohydrates (glucose, fructose, sucrose, high fructose corn-syrup (HFCS)), for example through soft drinks [7,8]. In addition, soft drink consumption has a limited effect on overall satiety often resulting in additional caloric intake and subsequent ‘energy-overload’ in turn promoting metabolic stress [9,10]. This is corroborated by several studies in humans that have shown a link between soft drink consumption and obesity, insulin resistance and NAFLD [11,12,13,14]. More specifically, the addition of a fructose/corn-syrup based ‘soft drink component’ to a high fat diet has also been shown to accelerate NAFLD/NASH in some experimental animal models, consequently promoting the use of HFCS in preclinical disease modeling [15,16,17,18,19,20].

In contrast to simple carbohydrates, the physiological response to starch is underappreciated in many in vivo studies. Unlike fructose and glucose (the primary components of HFCS) starch is a polysaccharide and a major nutritional constituent of most human diets, as well as carbohydrate source in various rodent diets [8,21,22]. While starch has not been directly associated with the development of NAFLD, starches with a high glycemic index have been linked to obesity, diabetes and hyperlipidemia in humans, supporting an effect on metabolism [23,24,25]. Consequently, the starch content of control diets applied in preclinical modeling might induce a metabolic state with little relevance to a healthy human control and—unintentionally—reduce the value of comparisons. For the guinea pig NAFLD/NASH model, a low-fat high-starch (LF-HSt) diet promotes a healthy liver phenotype, however guinea pigs do not display differences in weight or glucose tolerance compared to a HFD [26,27,28,29,30]. To investigate putative differences in a more metabolically relevant control group, a diet low in fat and starch was included (LF-LSt). Moreover, as current clinical guidelines dictate a diet change to a low cholesterol/low saturated fat diet such as the Mediterranean diet or a hypocaloric diet in addition to therapeutic treatment, the potential use of improper controls in preclinical studies may overshadow findings of therapeutic effects, seriously limiting the ability to identify new drug candidates [31,32]. 

Here, we investigate the role of different carbohydrate sources by including three HF diet groups of which two were given free access to HFCS in their drinking water. The effects of the HF diets with or without sugar water were compared to two low-fat groups (LF) one with high amounts of starch (LF-HSt) and one with low amounts of starch (LF-LSt), the latter hereby targeting a low-fat/low-carbohydrate diet with proposedly minimal effect on metabolism.

## 2. Materials and Methods

### 2.1. Animals

All animal experimentation was approved by the Animal Experimentation Inspectorate under the Danish Ministry of Environment and Food, and in accordance with European legislation of Animal Experimentation 2010/63/EU. The study was conducted in accordance with the ARRIVE guidelines [33].

Forty female Hartley guinea pigs weighing 401–450 g (Charles River Laboratory, Lyon, France) were tagged with an XS 1.4 mm subcutaneous microchip (Evet, Haderslev, Denmark) upon arrival. In coherence with previous studies, this experiment was performed in female guinea pigs, as hierarchical fighting between males poses serious concerns for animal welfare. Following one week of acclimatization animals were weight-stratified into 5 groups (*n* = 8/group): low-fat low-starch (LF-LSt): 3.8% fat, 0% sucrose, 0% cholesterol, 13.4% starch, low-fat high-starch (LF-HSt): 4.3% fat, 0% sucrose, 0% cholesterol, 28.4% starch, or a high-fat sucrose diet (20% fat, 15% sucrose, 0.35% cholesterol, 7.9% starch) and receiving either tap water (HF) or HFCS supplemented drinking water (4.2% + HF): 4.2% (45% d-glucose, 55% d-fructose (Sigma Aldrich, St. Louis, MO, USA), or (8.4% + HF): 8.4% (19% d-glucose sugar, 23% d-fructose, 58% sucrose (Sigma Aldrich, St. Louis, MO, USA) (for more details on diet composition see Tables S1 and S2). To allow comparison to the 4.2% group, and as humans only consume around 50% of their added sugars as HFCS and 50% as sucrose [34], 42% of the sugar in the 8.4% group was HFCS while sucrose constituted the remaining 58%.

Group sizes were based on power calculations (power of 80%, *p* < 0.05), using variances based on our previous studies and the ability to observe a difference in OGTT AUC of 30%. All diets were chow based and produced by Ssniff Spezialdiäten (Soest, Germany. See Table S2 for detailed diet information).

The switch from acclimatization standard chow to experimental diets was achieved gradually over a 5-day period. Likewise, sugar water was introduced gradually following the diet change, with tap water being substituted with either 4.2 or 8.4% sugar water over a period of 7 days after which animals in the sugar water groups had ad libitum access to sugar water, and restricted access to normal water (150 mL/group/day) to ensure animal welfare. The LF-LSt group was pair-fed to the LF-HSt group ensuring lower calorie-intake in the LF-LSt group, whereas HF fed groups had ad libitum access to food. In an effort to limit additional starch intake, all animals had restricted access to hay. Daily food intake was calculated by subtracting the amount of food remaining with the amount given the previous day. Water intake was calculated in the same manner, also on a daily basis (due to daily fluctuations calculations of food, water and caloric intake is based on weekly averages). Body weights were measured once weekly.

### 2.2. Oral Glucose Tolerance Test (OGTT)

All animals were semi-fasted (allowing access to hay and water) for 12 h prior to testing. Testing was performed over the course of two days. To ensure unbiased sampling, animals were block randomized within groups based on weight, testing half the animals on day one and the other half the second day. On the day of testing, animals were administered 2 g/kg of a 150% glucose solution by oral dosing. In case of administration exceeding 3 min, the animal was excluded from the test. Blood samples were collected at 0 (baseline, prior to glucose administration), 30, 60, 90 and 120 min by puncturing the ear vein with a 27G needle and subsequently, glucose measurement was achieved on an Aviva Accu-chek Glucometer (Roche A/S Diagnostics, Hvidovre, Denmark), as described previously [35]. All sampling was carried out in duplicates. At week 16, three animals (two from the 8.4%, and one animal from the HF group), were excluded based on the 3 min administration criteria. 

### 2.3. Insulin Tolerance Test (ITT)

All animals were semi-fasted and randomized as described for OGTT. A dose of 0.5 U/kg insulin (Actrapid^®^ (Novo Nordisk A/S, Bagsværd, Denmark) was injected subcutaneously in the neck (27G needle). Blood samples were collected at time points 0 (baseline, prior to insulin administration), 15, 25, 50, 75 and 120 min and glucose measured as described for OGTT. All sampling was carried out in duplicate. For week 8, three animals were excluded (one animal from 8.4% + HF, one from HF and one from LF-LSt group). One animal was excluded from the ITT due to hypoglycemia (glucose levels below 2 mM) and was treated with oral glucose supplement. The two remaining animals were excluded due to inaccurate dosing (due to handling issues when fixating the animals, a small amount of insulin was unfortunately not injected).

### 2.4. Euthanasia and Sampling

At euthanasia animals were semi-fasted overnight before being pre-anaesthetized with 1.25 mL/kg Zoletil-mix (125 mg Tiletamin, 125 mg Zolazapam (Zoletil 50 Virbac Laboratories, Carros, France) + 200 mg xylazin (Narcoxyl vet 20 mg/mL; Intervet International, Boxmeer, Holland) + 7.5 mg butorphanol (Torbugesic vet 10 mg/mL; Scanvet, Fredensborg, Denmark). To ensure accurate dosing and isotonicity, the anesthetic mix was diluted 1:10 in isotonic NaCl and animals were dosed with 1.25 mL/kg body weight. Once anesthetized, animals were placed on isofluorane (3–5%) inhalation through a mask. Upon disappearance of inter-digital reflexes, intra-cardial blood was collected; for vitamin C, vitamin E, uric acid, malondialdehyde (MDA) analysis blood was collected in an EDTA coated 10 mL syringe, whereas samples for free fatty acids (FFA) and alkaline phosphatase (ALP) analysis were collected in sodium fluoride (NaF) and heparin coated microvettes (Sarstedt, Nümbrecht, Germany) respectively. For triglyceride (TG), aspartate aminotransferase (AST), alanine aminotransferase (ALT) and total cholesterol (TC) blood samples were collected in K3 EDTA coated microvettes (Sarstedt, Nümbrecht, Germany). Plasma was isolated by centrifugation at 2000× *g* for 4 min at 4 °C. The isolated plasma for FFA, ALP, TG, TC, ALT, AST was stored in Cobas cups (Sample cup micro 13/16, Roche Diagnostics, Mannheim, Germany) at −20 °C, until analysis on a Cobas 6000 (Roche Diagnostics, Berne, Switzerland), according to manufacturer’s instructions.

Plasma for vitamin C measurement was immediately stabilized in metaphosphoric acid before storing at −80 °C. Plasma samples for vitamin E, uric acid, and MDA were transferred to 1.5 mL Eppendorf^®^ tubes and stored at −80 °C, until analysis by high performance liquid chromatography as previously described [36,37,38].

### 2.5. Liver Samples

To retrieve liver samples, the whole liver was excised and briefly rinsed in ice-cold PBS. Six liver sections were then obtained from lobus sinister lateralis to limit variation, as previously described [39]. Four sections were immediately frozen on dry ice and stored at −80 °C for TG, TC, MDA, vitamin C, vitamin E, tetrahydrobiopterin (BH4), dihydrobiopterin (BH2) determination. The remaining two sections were fixed in 10% formalin in separate containers, one for histological staining and one for other purposes (not used in this study). Liver tissue for TG and TC analysis was prepared as previously described [40], and analyzed on a Cobas 6000 (Roche Diagnostics, Berne, Switzerland), according to manufacturer’s instructions. MDA, vitamin C, vitamin E, BH2 and BH4 were all determined by high performance liquid chromatography, as previously described [36,37,38,41,42].

### 2.6. Histology

Paraffin-embedded liver sections were sliced in sections of 2–4 µm and stained with hematoxylin and eosin (H&E) or Picro Sirius Red (PSR) with Weigert’s hematoxylin solution. To ensure reliable scoring, 10 random sections were selected for calculation of Cohen’s Kappa index [43,44,45]. The 10 sections were scored in a blinded manner, re-blinded and then re-scored. Cohen’s Kappa values were then calculated, and the observer was only allowed to proceed with the actual scoring if Kappa values ≥0.8 (steatosis, inflammation, ballooning, fibrosis). Scoring was performed in a blinded and randomized manner as previously described for the guinea pig model, and based on Kleiner et al. [28,46]. In short, steatosis, inflammation and hepatocellular ballooning was evaluated on H&E stained sections, fibrosis was evaluated on PSR stained sections. Steatosis was evaluated across the entire liver section and scored as 0 (<5%), 1 (5–33%), 2 (>33–66%), 3 (>66%). Lobular inflammation was scored in five separate lobules dispersed across the entire section. A lobule was defined as two portal areas and one central vein, with an inflammatory focus defined as three or more inflammatory cells in close proximity and scored as 0 (no foci), 1 (<2), 2 (2–4), or 3 (>4). Hepatocyte ballooning was evaluated across the entire section and scored as 0 (none), 1 (few), 2 (many). Fibrosis was evaluated across the entire section and scored as 0 (none), 1 (perisinusoidal or periportal), 2 (perisinusoidal and periportal), 3 (bridging), 4 (cirrhosis). NAFLD activity score (NAS) was derived from the cumulative sum of steatosis, inflammation and ballooning ranging from 0 to 8 [46].

### 2.7. Statistics

All statistical analyses were performed in the GraphPad Prism version 9.0.1 (GraphPad Prism software, La Jolla, CA, USA). All continuous normally distributed data with equal variances among groups were analyzed by a parametric test (either one-way ANOVA, two-way ANOVA with repeated measures, or mixed effects model), with Tukey’s test for multiple comparisons, and presented as means with standard deviation (SD). Upon deviations from normality, data were log transformed and re-analyzed, these data are presented as medians with 25th and 75th quartiles. If the data continued to deviate or were categorical, they were analyzed by a non-parametric Kruskal–Wallis with a Dunn’s test for multiple comparisons and presented as medians with 25th and 75th quartiles. If there were unequal variances between groups data were analyzed by Welch-ANOVA with a Dunnett’s test for multiple comparisons and presented as means with SD. Repeated measures two-way ANOVA with a Tukey’s test for multiple comparisons were performed for body weights OGTT and ITT. In case of missing values, a repeated measures mixed-effects analysis with Tukey’s test for multiple comparisons was performed instead.

## 3. Results

### 3.1. Diet Composition, Calorie Intake and Body Weight

**Figure 1:**
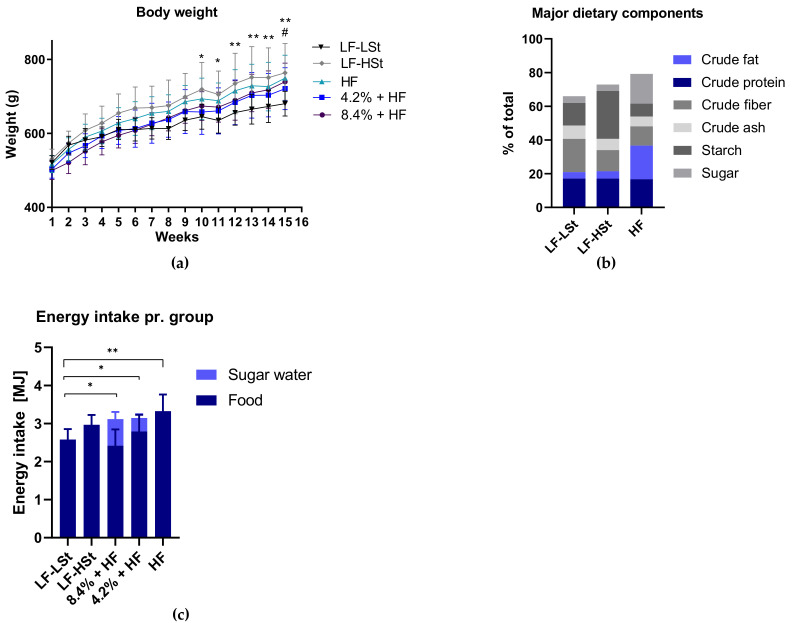
Body Weight and Energy Intake. (**a**) Body weights for each group are presented as means with SD, for each week. Data was analyzed using a mixed effects model with repeated measures with a Tukey’s test for multiple comparisons. *n* = 8 (**b**) Major dietary components presented in % (**c**) Energy intake in MJ for each group is presented as means with SD and analyzed using a one-way ANOVA with a Dunnett’s test for multiple comparisons. *n* = 13 average weekly intake pr. group during the study period. For LF-LSt vs. LF-HSt * *p* < 0.05, ** *p* < 0.01. for LF-LSt vs. HF ^#^ *p* < 0.05. LF: Low Fat, HSt: High Starch, LSt: Low Starch, HF: High Fat, MJ: Mega Joule.

Bodyweights and energy intake was monitored for all groups throughout the study period (Figure 1a,c). The main differences in the basic dietary components of the LF-LSt, LF-HSt and HF groups were crude fat, crude fiber, starch and sugar content (Figure 1b). Although the metabolizable energy in the HF diet (16.8 MJ/kg) is greater than in LF-HSt (12.6 MJ/kg) and LF-LSt (11.2 MJ/kg) diets (Table S1), the total energy intake of the HF animals was only higher compared to LF-LSt (Figure 1c). The total energy and sugar water intake of the sugar water groups (SW; 4.2% and 8.4%) was similar (Figure 1c and Figure S1a). Therefore, it is not surprising that the 8.4% group ingests ~50% more calories from sugar water compared to the 4.2% group (0.70 MJ and 0.36 MJ, respectively (Figure 1c)). As shown in Figure 1c, the increased calorie intake from the sugar water was accompanied by a concomitant decrease in food intake, in the 8.4% group compared to the 4.2% group corresponding to the surplus calories received from the sugar water (0.37 MJ). The calorie intake was poorly reflected in the body weights of the various groups (Figure 1a). From week 10 and on LF-HSt showed higher body weight compared to LF-LSt, despite similar calorie intake (Figure 1a,c). At week 15, the LF-HSt and the HF groups showed significantly higher body weights compared to LF-LSt.

### 3.2. Oral Glucose Tolerance Testing

**Figure 2:**
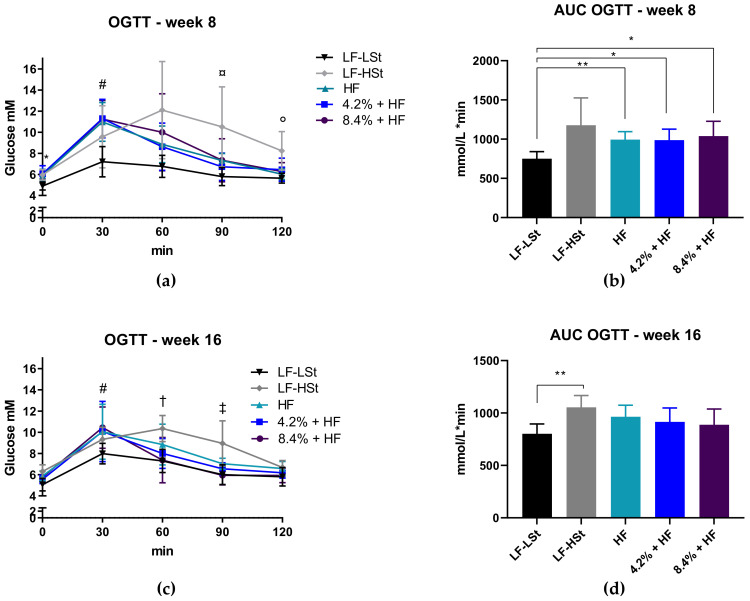
Oral Glucose Tolerance Tests. (**a**) Oral glucose tolerance test at week 8. Data are presented as means with SD and square root transformed data were analyzed by a repeated measures two-way ANOVA, and a Tukey’s test for multiple comparisons. *n* = 8 (**b**) Area under the curve for each group. Data are represented as means with SD, and analyzed by Welch ANOVA with a Dunnett’s test for multiple comparisons. *n* = 8 (**c**) Oral Glucose tolerance test week 16. Data are represented as means with SD, and analyzed by mixed effects model with repeated measures, and a Tukey’s test for multiple comparisons. *n* = 6–8 (**d**) Area under the curve for each group. Data are presented as means with SD and analyzed by a one-way ANOVA, with a Dunnett’s test for multiple comparisons. *n* = 6–8. For (**a**,**c**): * LF-LSt different from all, ^#^ LF-LSt different from all except LF-HSt, ^¤^ LF-LSt different from HF and LF-HSt, ° LF-HSt different from HF and LF-LSt, ^†^ LF-HSt different from all except HF, ^‡^ LF-HSt different from all. *p* < 0.05. For (b,d): * *p* < 0.05, ** *p* < 0.01. LF: Low Fat, HSt: High Starch, LSt: Low Starch, HF: High Fat, AUC: Area Under Curve, OGTT: oral glucose tolerance test, Min: Minutes.

To assess effects on glucose homeostasis (as a measure of metabolic state), OGTT and ITT was performed after 8 and 16 weeks on diets. All HF fed animals displayed altered glucose tolerance after 30 min compared to LF-LSt animals on both investigated time-points (Figure 2a,c). In contrast, at the 8 week time-point the LF-HSt group displayed altered glucose tolerance compared to LF-LSt at the 90 min time point and compared to LF-LSt and HF at 120 min (Figure 2a). This was not as clear after 16 weeks, as the LF-HSt group differed from all groups except HF already at the 60 min time point, and differed from all groups at the 90 min time point. The increased glucose levels in the LF-HSt group were also reflected in the AUCs for week 16 (Figure 2d), where only LF-HSt was different from LF-LSt. In contrast, the AUCs from week 8 revealed differences between LF-LSt and all HF fed groups, but no difference between any of the HF diet groups, or the LF-LSt and LF-HSt. This might seem counterintuitive as the LF-HSt group displayed the highest overall mean, however due to the large SD in this particular group, results did not reach statistical significance (*p*-value = 0.075). Insulin tolerance tests revealed decreased insulin sensitivity in the LF-HSt only after 8 weeks. No differences were observed after 16 weeks (Figure S2).

### 3.3. Plasma Biochemical Markers

In support of the dietary cholesterol supplementation (Table S1), plasma TC levels were decreased in the LF-LSt group compared to all other groups (*p* < 0.001), which was already evident after 8 weeks of HF feeding (Table S5). Likewise, all HF diet groups displayed higher levels compared to LF-HSt (*p* < 0.001) (Table 1). FFA and TG showed a slightly different picture with higher levels in LF-HSt compared to LF-LSt and 8.4% + HF (*p* < 0.05). Liver damage marker AST was increased in all HF groups compared to both LF groups (*p* < 0.05), at both 8 and 16 weeks (Table S5 and Table 1). This was also seen for ALT in the HF and the 8.4% group at 16 weeks, whereas ALT in 4.2% animals only differed from LF-LSt (*p* < 0.05). Plasma α-Toc levels were increased in all HF groups compared to both LF groups, except for 8.4%, which was only increased compared to LF-LSt. Vitamin C and Uric acid levels were decreased in the HF group (*p* < 0.05, Uric acid and vitamin C), and in sugar water groups (*p* < 0.001, vitamin C) compared to LF-LSt. Plasma ALP levels were lower in the HF group compared to LF-LSt and both sugar water groups (LF-LSt vs. HF *p* < 0.01, HF vs. sugar water groups *p* < 0.05). There was no effect on MDA and γ-Tocopherol levels between groups (Table 1).

### 3.4. Liver Status

**Figure 3:**
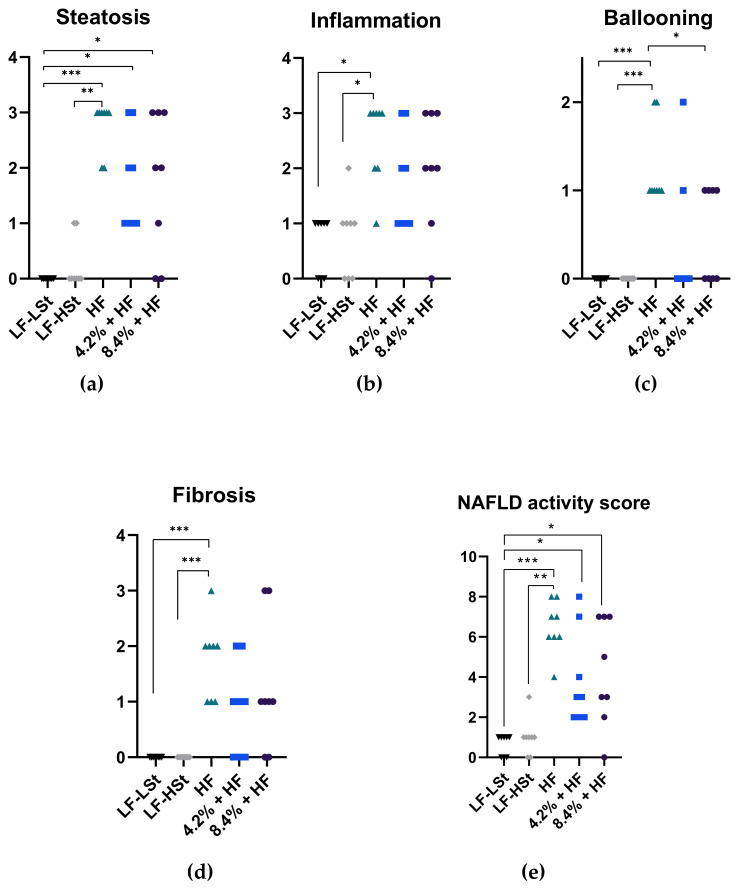
Histopathological scoring. Data are represented as individual scores with medians. (**a**) Steatosis scores for all groups on a scale of 0–3 (**b**) Inflammation scores for all groups on a scale of 0–3 (**c**) Ballooning scores for all groups on a scale of 0–2 (**d**) Fibrosis scores for all groups on a scale of 0–3 (**e**) Cumulative NAFLD activity score on a scale of 0–8. Scoring was performed as previously described [28]. The data was analyzed using a non-parametric Kruskal–Wallis with a Dunn’s multiple comparisons test. * *p* < 0.05, ** *p* < 0.01, *** *p* < 0.001. *n* = 8. LF: Low Fat, HSt: High Starch, LSt: Low Starch, HF: High Fat.

**Figure 4:**
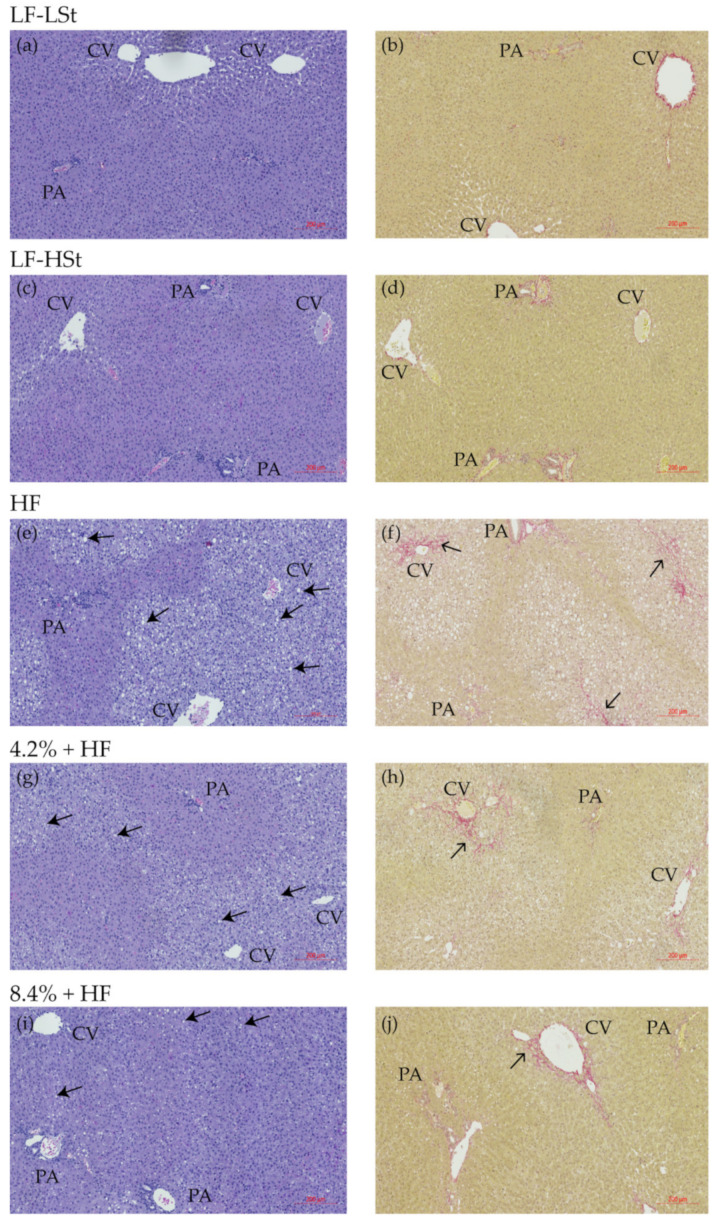
Representative histological images. (**a**,**c**,**e**,**g**,**i**) Hematoxylin and eosin stain. Scale bar shows 200 µm. (**b**,**d**,**f**,**h**,**j**) Picro Sirius Red stain. Scale bar shows 200 µm. Solid arrows indicate lipid vacuoles, and open arrows indicate fibrosis (in red). CV: central vein, PA: portal area, HF: high fat, LF: low fat, HSt: high starch, LSt: low starch.

To determine liver health status, biochemical markers and histopathology was assessed for all animals at week 16 (Table 2, Figure 3 and Figure 4). Liver TG and TC content was lower in the LF-LSt group compared to all other groups (*p* < 0.001, TC LF-LSt vs. LF-HSt *p* < 0.01). Liver TC was also significantly lower in LF-HSt compared to all HF groups (*p* < 0.001), whereas there was no difference between liver TG in LF-HSt and HF groups (Table 2). This was reflected in the steatosis score, as two animals in the LF-HSt group had a grade 1 steatosis, compared to LF-LSt where all animals displayed a grade 0. The steatosis score also revealed less advanced steatosis in both sugar water groups (4.2%: median grade 1.5, 8.4%: median grade 2), compared to the HF group (grade 3) (Figure 3); representative histological images depicted in Figure 4. Additionally, there was no difference in inflammation, ballooning or fibrosis between LF groups and the sugar water groups. This was in contrast to the HF group, which displayed higher scores in inflammation (*p* < 0.05), ballooning (*p* < 0.001) and fibrosis (*p* < 0.001) compared to both LF groups. There were no differences among markers of oxidative stress, apart from vitamin C levels, which were reduced in all groups compared to LF-LSt (*p* < 0.001) (Table 2). 

## 4. Discussion

The present study demonstrates significant differences in the response to various calorie sources in the guinea pig NAFLD/NASH model. Sugar water did not increase overall energy intake significantly and, consequently, led to a different hepatic phenotype and disease severity compared to high fat counterparts. Moreover, though curves progressed differently, all high fat diets and the high starch diet had a significant effect on the ability to maintain glucose homeostasis. Considering the variability of experimental diets also with regards to the composition of control diets, these findings raise awareness towards an important translational aspect of diet-induced NAFLD/NASH modeling.

Contrary to expectations, increased weight gain in sugar water groups was not observed, but is readily explained by the total energy intake matching HF counterparts. It should be kept in mind that values of metabolizable energy levels in feed constituents are not available for guinea pigs and the calculated levels of metabolizable energy are therefore derived from studies in rats and mice, hence might not be completely accurate (communication with ssniff [47]). In response to an increased energy intake from sugar water, guinea pigs in the 8.4% and 4.2% + HF groups decreased their caloric intake from food in a dose dependent manner. This highlights the ability of the guinea pig to fine tune their caloric intake, in line with previous findings in this species [28], and is also corroborated by Mock et al., who showed decreased food intake of Sprague-Dawley rats in response to either fructose, sucrose or HFCS addition to the drinking water [48]. Furthermore, Kohli et al. reported similar energy intake in C57Bl/6 mice fed a HF diet and mice fed a HF diet with 4.2% HFCS supplemented drinking water [17]. Although data specifying food and water intake was not available for this study, the lack of additional consumption of calories in the HF + HFCS group clearly indicates caloric compensation in this strain of mice [17]. The caloric compensation is in contrast to several human studies, where caloric intake in liquid form has a limited effect on satiety and food intake, hereby advancing excessive energy intake [9,10,49]. Thus, in rats, guinea pigs and mice, the effects of an added soft drink component on satiety and food intake are not directly comparable to what is seen in humans, potentially affecting the translational value of studies including these types of dietary regimes.

More than 50% of patients with type 2 diabetes also have NAFLD, making insulin resistance an important co-morbidity of NAFLD pathogenesis. In a study by Podell et al. using a high fat high carbohydrate diet (30% calories from fat, 52% calories from carbohydrates in the form of refined sugar), guinea pigs showed glucose and insulin intolerance as well as increased insulin levels compared to controls (3% fat, 18% protein, 55% complex carbohydrates, 10.5 MJ/kg) [50]. In contrast to these findings, we have not previously been able to measure insulin resistance in the guinea pig model for NAFLD/NASH [28,40]. However, the control diet in the study of Podell and coworkers had a lower energy content (10.5 MJ/kg) compared to the LF-HSt (12.6 MJ/kg, Table S1) diet used as a control diet for this and previous studies in our group [26,28,29,30,40,51]. Considering the lower energy content as well as the higher amount of refined sugar in the high fat diet, the current study explored if insulin resistance could be induced by HFCS supplements in the drinking water while ensuring comparison to a low-calorie low-starch control group (LF-LSt, 11.2 MJ/kg). The OGTT displayed increased glucose response in all HF groups at the 30 min time point compared to LF-LSt, indicating a significantly altered glucose homeostasis, though not a direct measure of insulin resistance. These findings were not supported by insulin tolerance testing that revealed no differences between LF-LSt and HF groups. The recorded discrepancies could indicate a decreased insulin response in HF animals upon subjection to an oral glucose load, rather than peripheral insulin resistance. 

While all HF groups resemble the general pattern of the LF-LSt group, the LF-HSt group displayed a different concourse with a delayed peak time and prolonged glucose clearance. A delayed peak time in OGTT in humans is associated with diabetes, increased HbA1c and decreased insulin sensitivity and secretion [52,53]. Delayed glucose absorption might contribute to a delay in peak-time. However, a previous study in diabetic rats demonstrated increased glucose transport, likely as a result of intestinal hypertrophy with a concomitant increase in SGLT1 expression in intestinal epithelial cells, compared to non-diabetic controls [54]. Another study assessing human intestinal glucose absorption in patients with type 2 diabetes, observed no difference in intestinal absorption between patients with diabetes and healthy controls [55]. This supports that the recorded delayed glucose peak in the LF-HSt group could be due to decreased insulin sensitivity and secretion. In addition, insulin tolerance testing revealed decreased insulin response in the LF-HSt group after 8 weeks, albeit this finding was not replicated at the 16-week time point (Figure S2). Accordingly, in this study high fat with or without sugar water supplementation and high starch diets lead to glucose intolerance, and only high starch appears to affect insulin tolerance. This finding could be attributed to the increased content of high glycemic index starch in the LF-HSt diet compared to the LF-LSt diet (Table S2), indicating that the amount of starch in control diets, either high or low glycemic, should be carefully considered. However, to establish a clear connection between starch and insulin resistance, further studies are needed. Regrettably, despite intensive efforts, attempts to measure insulin concentrations in guinea pig plasma samples have not been successful due to an absence of reliable commercially available detection methods for this species, thus limiting the ability to confirm insulin resistance in the current study. In addition, assessment of cellular signaling at a gene or protein level, e.g., the insulin receptor (INSR), insulin receptor substrate (IRS1) or GLUT4 (SLC2A4) in adipose and hepatic tissues, will be interesting for future studies investigating the cellular mechanisms underlying the current findings of metabolic dysregulation.

Corresponding to previous findings in the guinea pig NAFLD/NASH model, plasma TG and FFA levels (*p* < 0.05) were increased in the LF-HSt group, compared to the LF-LSt and the 8.4% + HF group. In line with these findings, a study with Sprague-Dawley rats assessing the effect of high or low glycemic index starches, demonstrated higher TG and FFA plasma levels in animals fed the high glycemic index starch compared to low glycemic index starches [56]. In a Chinese population study, the dietary habits and association with metabolic disorders were assessed for 4.154 randomly sampled participants (excluding individuals with cancer, type I diabetes, metabolic syndrome, or pregnant women) revealed high carbohydrate intake from starchy foods to be associated with increased risk of hyperlipidemia (RR: 1.73, 95% CI: 1.05–3.35) [23]. In the present study, plasma TG levels were positively correlated with body weight in the LF-HSt group, whereas hepatic TG levels showed an inverse correlation (Figure S3a,b), indicating that intake of high glycemic index starch is not stored in the liver but rather enters the circulation, and becomes stored in extrahepatic tissues. Consequently, this study shows the importance of considering the amount of starch used in the “control” diet and indicates a possible effect on hyperlipidemia, which may result in inclusion of a control group with little relevance to healthy individuals. The diets included in the current study all contain starch and animals were allowed access to hay in accordance with animal welfare legislation, preventing the evaluation of an isolated effect of starch. Though an inclusion of a non-starch group would be helpful in this aspect, an abstinence of dietary starch holds little relevance for both animal and human diets, and would not possess any significant translational value.

Ascorbate is a major free radical scavenger in the blood. Like humans, guinea pigs are unable to synthesize vitamin C, and are dependent on dietary supplementation. All groups displayed lower plasma and liver vitamin C levels compared to LF-LSt. This was in direct contrast to plasma α-Tocopherol levels, which were increased in all HF diet fed groups, in line with previous findings [30]. This increase is most likely due to the lipophilic nature of α-tocopherol and its facilitated uptake with dietary fat [57]. Low plasma vitamin C and a concomitant decrease in liver vitamin C levels could be an indication of increased oxidative stress and antioxidant usage in these animals. However, apart from its antioxidative capacity vitamin C is involved in a number of other processes, such as collagen synthesis cholesterol metabolism and proinflammatory signaling, which may contribute to disease progression [58]. Lower vitamin C status has previously been observed in both individuals with obesity and in guinea pigs on high fat diet [59,60]. It could also be argued that this is due to the higher food intake in the LF-LSt group. However, the observed differences persisted when accounting for food intake in each group (Figure S1b, Table S3).

While the role of simple carbohydrates in the development of NAFLD is well documented [61,62,63] the role of other carbohydrate sources, such as starch is less clear. In a study of obese children, isocaloric replacement of fructose with starch for 9 days improved liver steatosis, visceral fat and de novo lipogenesis, suggesting starch as a preferred carbohydrate source compared to fructose, albeit this was not compared to a low carbohydrate replacement diet [64]. A western/cafeteria diet, like the HF diet used in our study, has been shown to be a superior inducer of steatosis compared to HF diet alone, in C57BL/6 mice [65]. Furthermore, fructose alone or in combination with glucose (as HFCS) (13% *w/v*, administered through the drinking water) induced steatosis in rats on a control diet [48]. In the current study, increasing the amount of calories derived from simple sugars, did not seem to have an added effect on hepatic lipid accumulation in NAFLD as neither steatosis score nor liver triglycerides were increased in the sugar water groups compared to the HF group. However, liver TG was increased in the LF-HSt group compared to the LF-LSt, and two animals in the LF-HSt group displayed a mild degree of hepatic steatosis, a phenomenon that has been described previously for the guinea pig model and likely due to biological variation [29]. Regardless, excluding the two steatotic animals from the LF-HSt group did not affect the statistical conclusions of increased hepatic TG levels compared to LF-LSt, but resulted in significantly lower TG levels compared to all HF groups (Table S4). The findings from this study indicates a small increased risk of hepatic fat accumulation in guinea pigs subjected to a high starch diet. However, since the LF-LSt group and LF-HSt diets were not isocaloric, it cannot be ruled out that calorie excess itself may contribute to the increased hepatic lipid deposition. 

Hepatic histology revealed a milder phenotype in the sugar water groups, where ballooning, inflammation and fibrosis was not significantly different from low fat groups, indicating that HF alone was more potent in inducing advanced NASH. This reduction in disease phenotype is likely due to the recorded ‘energy-calibration’ in the guinea pigs, leading to a reduced feed intake in both sugar-water groups, hence reducing the hepatic lipid and cholesterol burden and consequent NASH progression. This is in contrast to a study in C57Bl/6 mice, where HFD + HFCS was found to be more effective in inducing inflammation and fibrosis compared to HFD alone [17]. In addition, increased liver weights were recorded in mice fed an ALIOS diet (a diet high in trans-fat supplemented with 4.2% HFCS in the drinking water) with HFCS supplement rather than ALIOS diet alone, and liver triglycerides showed a tendency to be increased when omitting HFCS from the diet, albeit this difference was not significant [19]. However, in both studies, only mild (3/6 had no fibrosis, 2/6 had a grade 1 fibrosis, and 1 animal had a grade 2 fibrosis) or no fibrosis was present in the animals [17,19]. This may be explained by the lack of cholesterol in their experimental diets, as cholesterol is generally considered a critical factor in the development of advanced liver pathology [5,6,66].

## 5. Conclusions

In this study, we show the limited translational value of adding a soft drink component to an experimental diet and highlight potential undesirable effects of using a high starch control diet. Supplementation of HFCS in the drinking water in addition to a HF, did not exacerbate NAFLD progression but instead reduced feed intake in a dose-dependent manner, consequently reducing inflammation, hepatocyte ballooning and fibrosis and indicating that fat and cholesterol, were superior inducers of advanced NAFLD. In addition, consideration should be paid to putative effects on metabolic phenotype e.g. when choosing a low-fat control diet. Finally, this study shows that guinea pigs on a HF develop increased weight gain, glucose intolerance and NASH with fibrosis within 16 weeks compared to a LF-LSt diet, presenting an animal model recapitulating a large part of the NAFLD disease spectrum observed in humans.

## Figures and Tables

**Table 1:** Plasma biochemical markers.

	LF-LSt	LF-HSt	HF	4.2% + HF	8.4% + HF
FFA mmol/L ^1^	0.57 (0.5–0.65)	0.77 (0.67–0.98) *	0.58 (0.51–0.65)	0.74 (0.7–0.75)	0.54 (0.45–0.62) ^#^
TG mmol/L ^1,3^	0.61 (0.5–0.62)	1.51 (1.27–2.12) ***	0.79 (0.66–0.97)	0.83 (0.74–0.92)	0.81 (0.62–0.89) ^#^
TC mmol/L ^1,2^	0.76 (0.68–0.84)	1.22 (1.03–1.45) ***	8.25 (5.85–13.41) ***^,###^	9.20 (7.69–14.48) ***^,###^	6.13 (4.29–10.14) ***^,###^
AST U/L ^1^	43.25 (29.18–62.03)	55.35 (38.03–118.2)	479.7 (218.3–580.7) ***^,###^	219.1 (105.4–435.7) **^,#^	275 (86.75–467.7) **^,#^
ALT U/L ^1^	30.15 (22.98–44.08)	35.5 (30.75–43.68)	81.25 (62.95–87.73) ***^,##^	50 (42.3–73.03) *	53.9 (47.13–73.45) **^,#^
ALP U/L	75.88 ± 10.20	64.38 ± 7.21	53.25 ± 9.88^**^	70.75 ± 13.99 ^†^	72.43 ± 15.09 ^†^
MDA µmol/L	0.42 ± 0.15	0.66 ± 0.19	0.57 ± 0.19	0.58 ± 0.15	0.51 ± 0.2
α-Tocopherol µmol/L ^1^	1.52 (1.34–1.61)	3.16 (1.64–3.99)	10.48 (7.2–16.68) ***^,###^	9.13 (6.78–15.17) ***^,###^	5.86 (3.57–9.74) ***
γ-Tocopherol µmol/L ^2^	0.03 ± 0.02	0.04 ± 0.03	0.07 ± 0.06	0.16 ± 0.1	0.04 ± 0.02
Total Vitamin C µM ^1^	42.76 (35.90–62.70)	21.70 (18.29–27.80) ***	27.20 (21.36–30.50) **	22 (18.63–26.53) ***	21.02 (17.94–24.64) ***
Uric acid µM ^1^	15.24 (12.72–20.49)	13.88 (13.23–14.99)	11.03 (10.09–13.56) *	12.50 (11.38–13.53)	13.66 (10.76–15.15)

Data are presented as means ± SD and Q25-Q75 values in brackets. Data was analyzed using a one-way ANOVA. ^1^ Log transformed data were analyzed by one-way ANOVA with a Dunnett’s test for multiple comparisons, and are presented as medians with quartiles in brackets. ^2^ Data was analyzed using a Welch ANOVA. ^3^ Data was analyzed using a Kruskal–Wallis test. Different from LF-LSt * *p* < 0.05, ** *p* < 0.01, *** *p* < 0.001, different from LF-HSt ^#^ *p* < 0.05, ^##^ *p* < 0.01, ^###^ *p* < 0.001, different from HF ^†^ *p* < 0.05. *n* = 8. LF: Low Fat, HSt: High Starch, LSt: Low Starch, HF: High Fat, FFA: Free Fatty Acids, TG: Triglycerides, TC: Total Cholesterol, AST: Aspartate Aminotransferase ALT: Alanine Aminotransferase, ALP: Alkaline Phosphatase, MDA: Malondialdehyde.

**Table 2:** Liver biochemical markers.

	LF-LSt	LF-HSt	HF	4.2% + HF	8.4% + HF
TG µmol/g ^1^	6.64 (4.00–8.95)	23.74 (19.35–71.76) ***	53.45 (36.67–58.90) ***	46.21 (41.52–49.21) ***	38.58 (30.24–56) ***
TC µmol/g ^1,2^	5.61 (4.66–6.38)	8.26 (7.61–8.56) **	35.03 (32.92–37.17) ***^,###^	29.62 (23.46–36.28) ***^,###^	27.33 (16.55–36.66) ***^,###^
α-Tocopherol µmol/g ^1^	3.35 (2.85–6.13)	3.1 (1–4.93)	2.1 (1.6–3.3)	2.25 (0.95–7.75)	2.05 (1.13–3.13)
BH2/BH4 µmol/g	0.2 ± 0.19	0.28 ± 0.16	0.19 ± 0.12	0.2 ± 0.11	0.29 ± 0.14
MDA nmol/g	140.3 ± 43.92	149.4 ± 64.83	130 ± 56.14	155.6 ± 59.47	180.3 ± 70.66
Total Vitamin C ^1^ µM	1810 (1567–2290)	1255 (1053–1471) ***	912.5 (820.9–1017) ***	962.3 (831.4–1023) ***	926.67 (873.6–1048) ***
	LF-LSt	LF-HSt	HF	4.2% + HF	8.4% + HF

Data are presented as means ± SD and Q25-Q75 values in brackets. Data was analyzed using a one-way ANOVA. ^1^ Log transformed data were analyzed by one-way ANOVA with a Dunnett’s test for multiple comparisons and presented as medians with quartiles in brackets. ^2^ Data was analyzed using a Welch ANOVA. Different from LF-LSt ** *p* < 0.01, *** *p* < 0.001, different from LF-HSt ^###^ *p* < 0.001. *n* = 8. LF: Low Fat, HSt: High Starch, LSt: Low Starch, HF: High Fat, TG: Triglycerides, TC: Total Cholesterol, BH2: Dihydrobiopterin, BH4: tetrahydrobiopterin, MDA: Malondialdehyde.

## Data Availability

The data presented in this article are available upon request from the corresponding author.

## Supplementary Materials

The following are available online at https://www.mdpi.com/article/10.3390/nu13082523/s1, Figure S1: Water and food intake pr. group, Figure S2: Insulin tolerance test, Figure S3: Correlation of body weight and triglyceride content in the LF-HSt group, Table S1: Major dietary components listed in %, Table S2: Detailed list of dietary components, Table S3: Total plasma and liver vitamin C levels corrected for food intake, Table S4: Liver TG levels upon exclusion of steatotic animals, Table S5: Plasma biochemical markers at week 8.
