# Supplementary material for: Differential Effects of Dietary Components on Glucose Intolerance and Non-Alcoholic Steatohepatitis
Source: Nutrients. 2021 Jul 23;13(8):2523. doi: 10.3390/nu13082523 (PMC8400624; doi:10.3390/nu13082523)
Supplement: Supplementary file 1 [file nutrients-13-02523-s001.zip › supp_figS1.pdf]

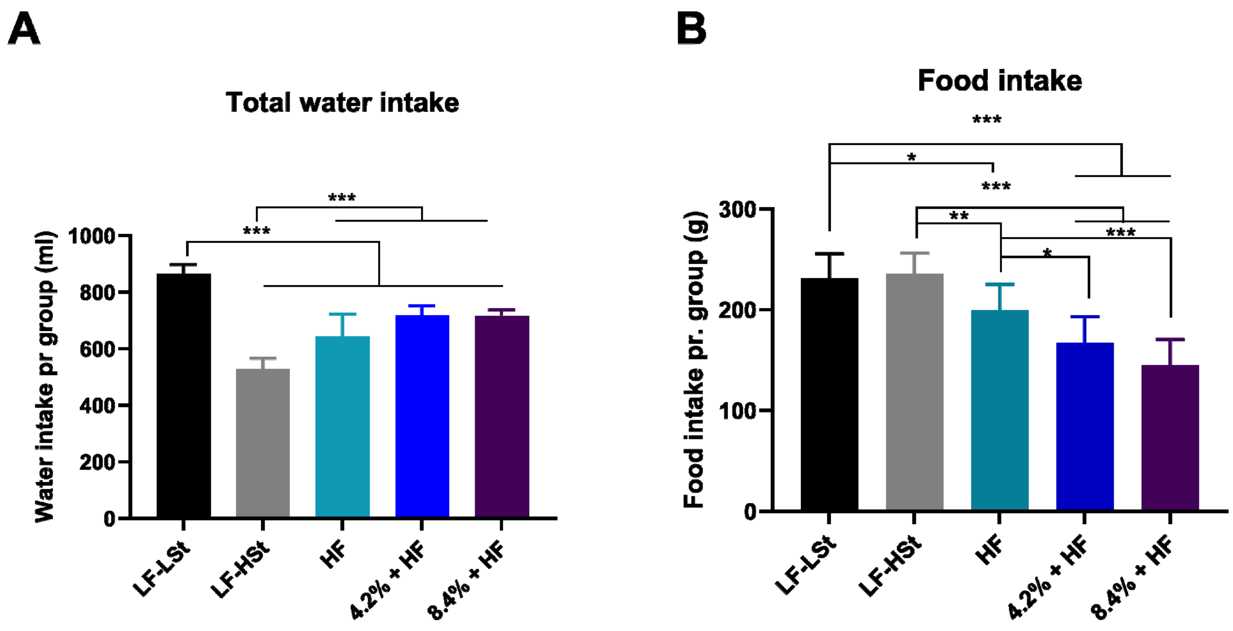

Figure S1. Water and food intake pr. group A Data are presented as medians with interquartile ranges and analyzed by one-way ANOVA on log transformed data with Tukey's test for multiple comparisons. **B** Data are presented as means with SD and analyzed by one-way ANOVA with a Tukey's test for multiple comparisons. n=13. *p<0.05, **p<0.01, ***p<0.001. LF: Low Fat, HSt: High Starch, LSt: Low Starch, HF: High Fat.
