# Supplementary material for: Differential Effects of Dietary Components on Glucose Intolerance and Non-Alcoholic Steatohepatitis
Source: Nutrients. 2021 Jul 23;13(8):2523. doi: 10.3390/nu13082523 (PMC8400624; doi:10.3390/nu13082523)
Supplement: Supplementary file 1 [file nutrients-13-02523-s001.zip › supp_figS2.pdf]

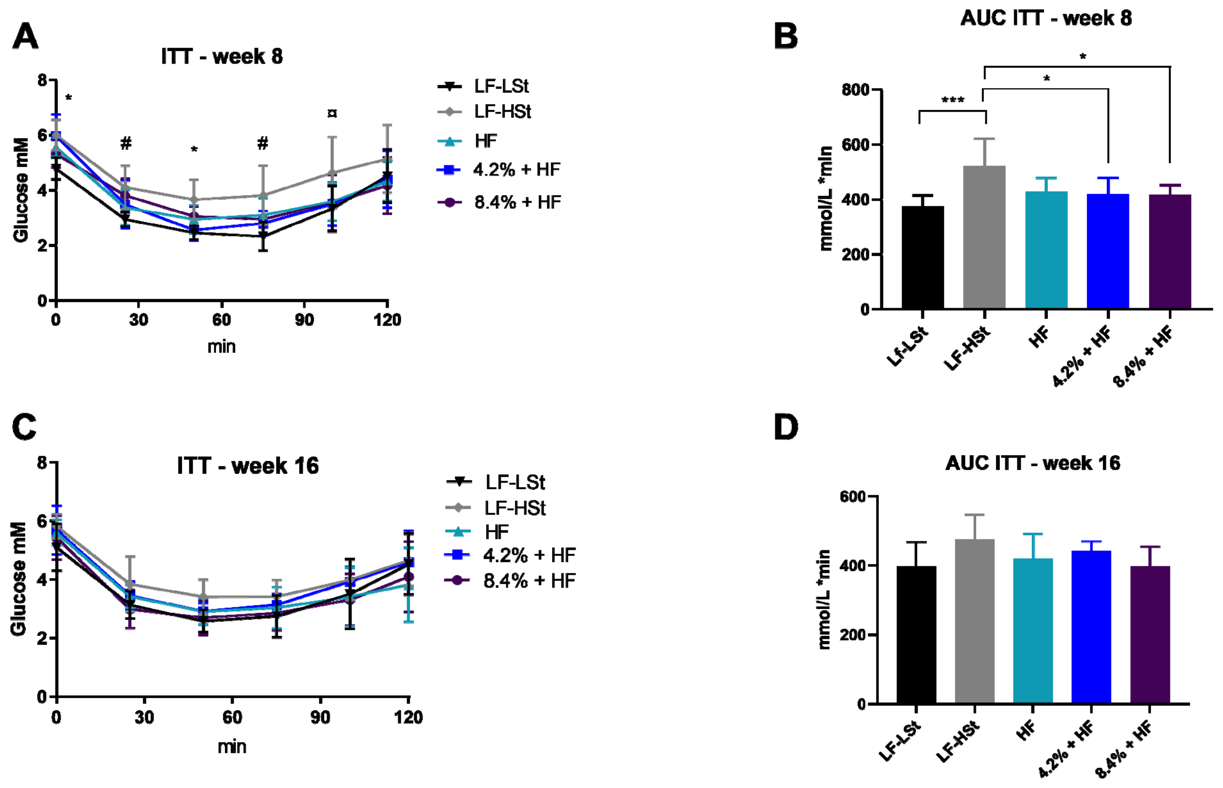

Figure S2. Insulin tolerance test. A Data are presented as means with SD and data were analyzed by a mixed effects model with repeated measures, and a Tukey's test for multiple comparisons. n=7-8 **B** Area under the curve for each group at week 8. Data are represented as means with SD, and log transformed data were analyzed by a one-way ANOVA with a Tukey's test for multiple comparisons. n=7-8 **C** Data are represented as means with SD, and analyzed by a repeated measures two-way ANOVA, and a Tukey's test for multiple comparisons. n=8 **D** Area under the curve for each group for week 16. Data are presented as means with SD and analyzed by a one-way ANOVA. n=8. *LF-LSt different from LF-HSt & 4.2% + HF, #LF-LSt different from LF-HSt, ¤LF-HSt different from LF-LSt and 4.2% + HF, †LF-HSt different from LF-LSt, 4.2% + HF and 8.4% + HF. P<0.05. LF: Low Fat, HSt: High Starch, LSt: Low Starch, HF: High Fat, AUC: Area Under Curve, ITT: Insulin Tolerance Test, Min: Minutes.
