# Supplementary material for: Differential Effects of Dietary Components on Glucose Intolerance and Non-Alcoholic Steatohepatitis
Source: Nutrients. 2021 Jul 23;13(8):2523. doi: 10.3390/nu13082523 (PMC8400624; doi:10.3390/nu13082523)
Supplement: Supplementary file 1 [file nutrients-13-02523-s001.zip › supp_figS3.pdf]

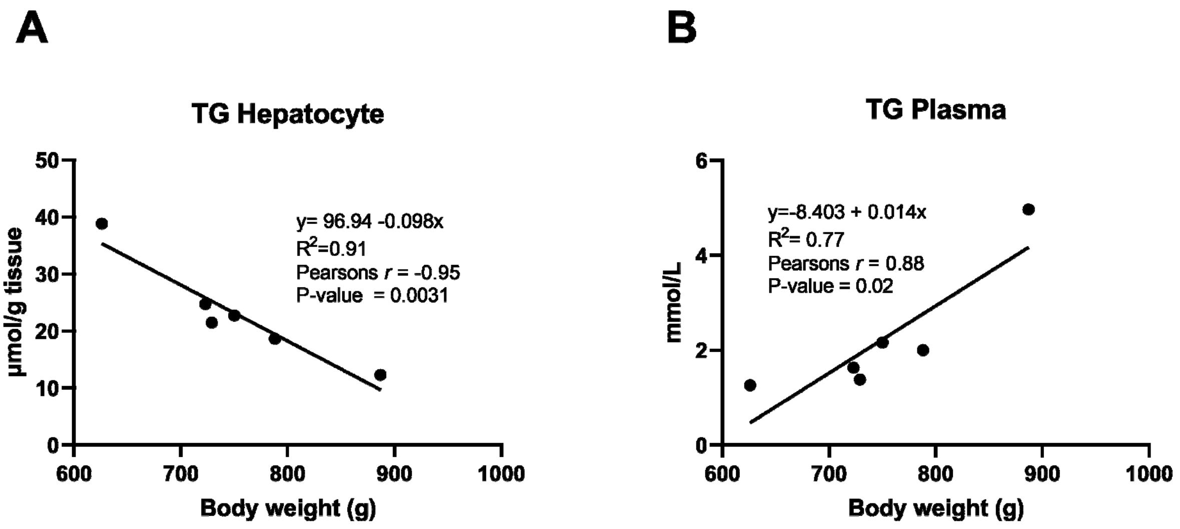

Figure S3. Correlation of body weight and triglyceride content in the LF-HSt group. Data are presented as individual values for each animal **A** Triglyceride levels in hepatocytes. Two animals are excluded due to development of steatosis, n=6 **B** Triglyceride levels in plasma, n=8. TG: Triglycerides.
