# Supplementary material for: Differential Effects of Dietary Components on Glucose Intolerance and Non-Alcoholic Steatohepatitis
Source: Nutrients. 2021 Jul 23;13(8):2523. doi: 10.3390/nu13082523 (PMC8400624; doi:10.3390/nu13082523)
Supplement: Supplementary file 1 [file nutrients-13-02523-s001.zip › table S5.pdf]

Table S5. plasma biochemical markers week 8

	**LF-LSt**	**LF-HSt**	**HF**	**4.2% + HF**	**8.4% + HF**
**TG mmol/L[1]**	0.25 (0.19-0.41)	0.95 (0.33-2.08)*	0.37 (0.34-0.62)	0.29 (0.21-0.31)$^{\#\#}$	0.29 (0.25-0.36)$^{\#\#}$
**TC mmol/L[1]**	0.49 (0.45-0.70)	0.9 (0.53-1.69)	8.7 (5.07-13.35)$^{***\#\#\#}$	5.3 (3.35-6.67)$^{***\#\#\#}$	3.72 (2.92-5.25)$^{***\#\#\#\dagger}$
**AST U/L[1]**	22.15 (17.88-42.3)	23.90 (16.95-48.55)	79.85 (55.75-159)$^{**\#}$	69.25 (42.43-183.6)$^{*\#}$	47.6 (34.68-87.85)
**ALT U/L[2]**	17.90 (13.33-(19.68)	18.90 (12.23-44.08)	25.85 (23.70-57.40)	20.40 (16.63-32.45)	20.50 (16.90-25.53)

Data are presented as medians with Q25-Q75 values in brackets. [1]Log transformed data were analyzed by one-way ANOVA with a Dunnett's test for multiple comparisons. [2]Data was analyzed using a Kruskal Wallis test. *Different from LF-LSt $^{*}p<0.05$, $^{**}p<0.01$, $^{***}p<0.001$, different from LF-HSt $^{\#}p<0.05$, $^{\#\#}p<0.01$, $^{\#\#\#}p<0.001$, different from HF $^{\dagger}p<0.05$, $^{\dagger}p<0.01$, $^{\dagger\dagger\dagger}p<0.001$ LF: Low Fat, LSt: Low Starch, HSt: High Starch, HF: High Fat, FFA: Free Fatty Acids, TG: Triglycerides, TC: Total Cholesterol, AST: Aspartate Aminotransferase, ALT: Alanine Aminotranferase, ALP: Alkaline Phosphatase.
