# Supplementary material for: Differential Effects of Dietary Components on Glucose Intolerance and Non-Alcoholic Steatohepatitis
Source: Nutrients. 2021 Jul 23;13(8):2523. doi: 10.3390/nu13082523 (PMC8400624; doi:10.3390/nu13082523)
Supplement: Supplementary file 1 [file nutrients-13-02523-s001.zip › Table_S1.pdf]

Table S1. Major dietary component are listed in %.

Component %	LF-LSt	LF-HSt	HF	4.2% + HF	8.4% + HF
**Crude protein**	17.1	17.1	16.7	16.7	16.7
**Crude fat**	3.8	4	20	20	20
**Crude fiber**	19.8	12	11.4	11.4	11.4
**Crude Ash**	7.9	6.7	6.6	6.6	6.6
**Starch**	13.4	28.4	7.7	7.7	7.7
**Sugar**	4	3.8	17.6	17.6	17.6
**Carbohydrates**	41.2	48.6	37.9	37.9	37.9
**Cholesterol**	0	0	0.35	0.35	0.35
**MJ/kg**	11.2	12.6	16.7	16.7	16.7
**Sugar water**				4.2%	8.4%
- HFCS-55 % of total	0	0	0	100%	42%
- Sucrose % of total				0	58%

LF: Low Fat, LSt: Low Starch, HSt: High Starch, HF: High Fat, SW: sugar water, MJ: Mega Joule, HFCS: High Fructose Corn Syrup. HFCS-55 is composed of 55% Fructose and 45% Glucose.
