# Supplementary material for: Differential Effects of Dietary Components on Glucose Intolerance and Non-Alcoholic Steatohepatitis
Source: Nutrients. 2021 Jul 23;13(8):2523. doi: 10.3390/nu13082523 (PMC8400624; doi:10.3390/nu13082523)
Supplement: Supplementary file 1 [file nutrients-13-02523-s001.zip › Table_S2.pdf]

Table S2. Detailed list of dietary components.

Product		LF-LSt	LF-HSt	HF
**Alfalfa**	%	33	22	22
**Wheat**	%	4	27.97	10
**Barley**	%	10	18	-
**Wheat Bran**	%	15.55	-	-
**Sucrose**	%	-	-	14.97
**Inulin**	%	3	-	-
**Cellulose (Lignocellulose)**	%	4	4.6	4.6
**Sunflower meal**	%	5	3	3
**Soybean meal**	%	10.2	12	6
**Soybeans (full fat)**	%	1	2.6	2.6
**Soybean concentrate**	%	-	2	12
**Corn gluten feed**	%	8	-	-
**Amino acids**	%	0.65	0.5	0.5
**Vitamin/trace element premix**	%	1	1	1
**Choline Cl**	%	0.3	0.3	0.3
**Vitamin C, stab. (StayC 35%)**	%	0.44	0.44	0.44
**Sugar beet pulp**	%	1.3	1	1
**Soybean oil**	%	1.2	2.1	0.5
**Cholesterol**	%	-	-	0.35
**Coconut oil, hydrogenated**	%	-	-	18
**Crude protein (=N x 6.25)**	%	17.1	17.1	16.9
**Crude fat**	%	3.8	4.3	20
**Crude fiber**	%	19.8	12.6	11.4
**NDF**[1]	%	32.3	23.2	-
**Crude ash**	%	7.9	6.7	6.6
**Starch**	%	13.4	28.4	7.9
**Sugar**	%	4	3.8	17.5

[1]NDF: Neutral Detergent Fiber; fiber fraction that is included in the CHO (NfE). [2]Atwater does not reflect correct ME of high fiber diets and ME for guinea pigs

**Carbohydrates (NfE)**	%	41.2	48.6	
**ME (Atwater)[2]**	MJ/kg	11.2	12.6	16.8
**Kcal% Protein**		26	23	17
**Kcal% Fat**		13	13	45
**Kcal% CHO**		62	64	38
**Fatty Acids, % in diet**				
**C8:0**		-	-	1.06
**C10:0**		-	-	0.86
**C12:0**		-	-	8.22
**C14:0**		0.01	0.02	3.53
**C16:0**		0.64	0.65	2.23
**C18:0**		0.11	0.14	2.32
**C20:0**		0.01	0.02	0.03
**C16:1**		0.02	0.02	0.01
**C18:1**		0.67	0.87	0.44
**C18:2**		1.89	2.22	0.96
**C18:3**		0.35	0.36	0.22
**Ascorbic acid**	Mg/kg	1500	1500	1500
