# Supplementary material for: Differential Effects of Dietary Components on Glucose Intolerance and Non-Alcoholic Steatohepatitis
Source: Nutrients. 2021 Jul 23;13(8):2523. doi: 10.3390/nu13082523 (PMC8400624; doi:10.3390/nu13082523)
Supplement: Supplementary file 1 [file nutrients-13-02523-s001.zip › Table_S3.pdf]

Table S3. Total plasma and liver Vitamin C levels corrected for food intake.

	**LF-LSt**	**LF-HSt**	**HF**	**4.2% + HF**	**8.4% + HF**
**Total Vitamin C** µM **(Plasma)**	43.63 (36.64-63.97)	21.70 (18.29-27.80)***	32 (25.13-35.88)*	30.14 (25.52-36.34)*	33.36 (28.48-39.11)$^{\#}$
**Total Vitamin C** µM **(Liver)**	1847 (1599-2337)	1255 (1053-1471)***	1074 (965.8-1196)***	1318 (1139-1402)**	1471 (1387-1664)†

Data are presented as medians with quartiles, and log transformed data were analyzed by a one-way ANOVA with Tukey's test for multiple comparisons. Different from LF-LSt $^{*}p<0.05$, $^{**}p<0.01$, $^{***}p<0.001$, different from LF-HSt $^{\#}p<0.05$, $^{\#}p<0.01$, $^{\#\#\#}p<0.001$. Different from HF $^{\dagger}p<0.05$ $^{\dagger\dagger}p<0.01$ $^{\dagger\dagger\dagger}p<0.001$. n=8. LF: Low Fat, HSt: High Starch, LSt: Low Starch, HF: High Fat.
