# Supplementary material for: Differential Effects of Dietary Components on Glucose Intolerance and Non-Alcoholic Steatohepatitis
Source: Nutrients. 2021 Jul 23;13(8):2523. doi: 10.3390/nu13082523 (PMC8400624; doi:10.3390/nu13082523)
Supplement: Supplementary file 1 [file nutrients-13-02523-s001.zip › Table_S4.pdf]

Table S4. Liver TG levels upon exclusion of steatotic animals.

	**LF-LSt**	**LF-HSt**	**HF**	**4.2% + HF**	**8.4% + HF**
TG µmol/g[1]	6.64 Q25:Q75: 4.00-8.95	22.10 Q25:Q75: 17.06-28.27***	53.45 Q25:Q75: 36.67-58.90***#	46.21 Q25:Q75 41.52-49.21***##	38.58 Q25:Q75: 30.24-56***##

Data are presented as medians with quartiles and log transformed data were analyzed by one-way ANOVA with Tukey's test for multiple comparisons. Different from LF-LSt *p<0.05, **p<0.01, ***p<0.001, different from LF-HSt #p<0.05, ##p<0.01, ###p<0.001. n=8. LF: Low Fat, HSt: High Starch, LSt: Low Starch, HF: High Fat, TG: Triglycerides.
